# Natural Fiber-Stabilized Geopolymer Foams—A Review

**DOI:** 10.3390/ma13143198

**Published:** 2020-07-17

**Authors:** Katharina Walbrück, Felicitas Maeting, Steffen Witzleben, Dietmar Stephan

**Affiliations:** 1 Department of Natural Sciences, Bonn-Rhine-Sieg University of Applied Sciences, von-Liebig-Str. 20, 53359 Rheinbach, Germany; felicitas.maeting@smail.bcw.h-brs.de (F.M.); steffen.witzleben@h-brs.de (S.W.); 2 Building Materials and Construction Chemistry, Department of Civil Engineering, Technische Universität Berlin, Gustav-Meyer-Allee 25, 13355 Berlin, Germany; stephan@tu-berlin.de

**Keywords:** geopolymer foam, thermal insulation material, natural fiber

## Abstract

The development of sustainable, environmentally friendly insulation materials with a reduced carbon footprint is attracting increased interest. One alternative to conventional insulation materials are foamed geopolymers. Similar to foamed concrete, the mechanical properties of geopolymer foams can also be improved by using fibers for reinforcement. This paper presents an overview of the latest research findings in the field of fiber-reinforced geopolymer foam concrete with special focus on natural fibers reinforcement. Furthermore, some basic and background information of natural fibers and geopolymer foams are reported. In most of the research, foams are produced either through chemical foaming with hydrogen peroxide or aluminum powder, or through mechanical foaming which includes a foaming agent. However, previous reviews have not sufficiently addresses the fabrication of geopolymer foams by syntactic foams. Finally, recent efforts to reduce the fiber degradation in geopolymer concrete are discussed along with challenges for natural fiber reinforced-geopolymer foam concrete.

## 1. Introduction

**Figure 1:**
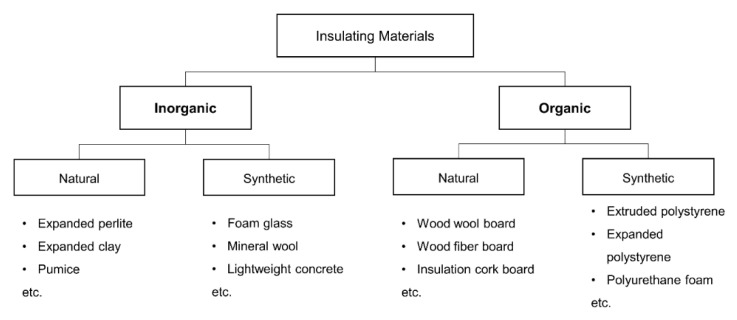
Classification of thermal insulation materials according to Reference [2].

According to the international energy agency, buildings are responsible for 28% (2018) of the global energy-related CO_2_ emissions, while heating is responsible for the largest share. However, thermal insulation reduces the heating and cooling demands of buildings and therefore reduces the energy demand [1]. In general, thermal insulating materials can be classified according to their raw materials into inorganic or organic and natural or synthetic materials (Figure 1).

The most common insulating materials, such as extruded or expanded polystyrene, mineral wool or lightweight concrete, are based on non-renewable resources like petroleum or cement [3,4]. Cement in particular is one of the largest producers of greenhouse gases and in the industry sector responsible for 26% of the CO_2_ emissions. Ordinary Portland cement (OPC) was the most used construction material worldwide in 2018, due to its high durability and low cost: approximately 4.1 billion metric tons of cement was produced [5,6]. However, OPC has a high embodied energy and a global warming potential of 0.6 metric tons CO_2_ (only for Germany in 2018) for every tonne of cement [7]. Therefore, the interest in the development of sustainable thermal insulating materials is growing. One alternative to cement-based insulation materials is foamed geopolymers. Previous studies have reported a reduction in CO_2_ emissions of up to 80% compared to OPC, due to the use of industrial waste or by-products in geopolymer binders [8,9,10,11]. These CO_2_ emission data are based on a life cycle assessment (LCA) according to ISO 14067 [12]. However, various factors influence the results of LCA studies. According to Teh et al., methodological problems such as setting a system boundary for the analysis, amounts of emissions from upstream supply chain processes or the use of country specific data leads to inaccuracies [8,9,10,11]. Furthermore, to improve the properties of the final geopolymers, additives like natural fibers or nanoparticles can be used [13,14]. Nanoparticles are sometimes used to enhance the mechanical properties and, in combination with natural fiber nanoparticles, reduce the degradation of the fibers, so better durability of the composites is achieved [14]. Natural fibers themselves have several positive characteristics, such as low cost, low density and a low thermal conductivity [13]. Therefore, this review paper focuses on foamed geopolymer systems that incorporate natural fibers and nanoparticles to improve the properties of the final geopolymer.

### 1.1. Geopolymers

#### 1.1.1. Geopolymerization

**Scheme 1:**
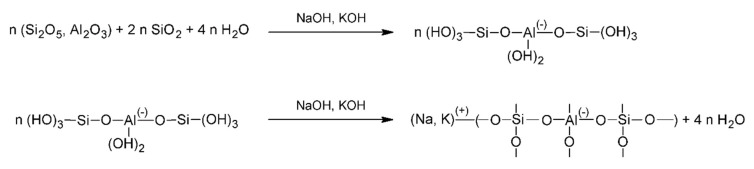
Hypothetical reaction mechanism for orthosialate ions according to Reference [20].

According to Davidovits, the synthesis of geopolymers can, in general, be described as a reaction of aluminosilicate with alkali polysilicates which leads to the formation of Si–O–Al bonds [15]. In contrast to the activation of high-calcium alkaline slags, which produce a calcium silicate hydrate-based gel with one dimensional silicone chains (with some substitution of Al for Si and Mg for Ca), geopolymers contain less calcium and create amorphous or semi-crystalline three-dimensional macromolecular structures. These structures can be divided, according to their monomer unit, into three different types: the poly(sialate) type (Si–O–Al–O–), the poly(sialate–sil–oxo) type (Si–O–Al–O–Si–O–), and the poly(sialate–disil–oxo) type (Si–O–Al–O–Si–O–Si–O–). The geopolymerization reaction leading to these structures is exothermic and believed to be the result of a dissolution, transportation or orientation process followed by a polycondensation (Scheme 1) [15,16,17,18,19].

According to Xu and van Deventer, the dissolution of silica and alumina requires the presence of alkali metal salts or hydroxides, which are also needed as catalysts for the condensation reaction [21]. The dissolution of aluminosilicates is very rapid at high pH values and results in the creation of a supersaturated solution [22,23,24]. It is most likely that the coordination of aluminum ions (IV- or VI-fold coordination) in the aluminosilicate structure of the raw material has an effect on the formation of the Si–O–Al bonds during the following reaction [21,25].

The first phase of the geopolymerization reaction is characterized by the formation of a highly reactive intermediate gel, which is a result of the co-polymerization of individual alumino and silicate species [21,26]. The oligomers in the aqueous phase continue to condensate and form large networks. Incorporated water is released during the polycondensation and remains within pores in the gel [22].

The reaction continues until a network is created, which consists of SiO_4_ and AlO_4_ tetrahedra linked by sharing all oxygen atoms [16]. Because of the IV-fold coordination of Al^3+^, metallic cations such as Na^+^, K^+^ and Ca^2+^ are needed to keep the structure’s neutrality [27]. The process occurs at low temperatures, typically at room temperature or below 100 °C [16].

#### 1.1.2. Raw Materials

In general, geopolymers consist of an aluminosilicate precursor and an alkaline activator. The aluminosilicate precursors must be rich in aluminum (Al) and silicon (Si). Therefore, the most common aluminosilicate precursors used for geopolymer synthesis are fly ash (FA), ground granulated blast-furnace slag (GGBFS) and metakaolin (MK).

Fly ash (FA) is an industrial waste or by-product generated in coal-fired power plants. During combustion processes, all non-flammable and mineral components are melted into mostly spherical and glassy particles. Afterward, approximately 99% of these particles are collected from the exhaust gas flow by an electrostatic filter [28,29,30]. These particles have a diameter in the range of 0.5 to 300 µm and are mainly composed of silicon dioxide (SiO_2_), aluminum oxide (Al_2_O_3_) and iron(III) oxide (Fe_2_O_3_). Besides these components, some secondary components such as Ca, Mg, Na and K oxides are also accumulated in the particles [31]. Fly ash can either be classified according to EN 197-1 [32] due to their amount of reactive calcium oxide into siliceous V (>10 wt. % CaO) and calcareous W (10 to 15 wt. % CaO) fly ash or according to ASTM C618-19 [33] into Class N (raw or calcined natural pozzolans), F (pozzolanic properties) and C (pozzolanic and cementitious properties). Hajimohammadi et al. and Al Bakri Abdullah et al. used fly ash with a high calcium oxide amount of 13.6 wt. % and 21.6 wt. % for the geopolymer synthesis. The high amount of CaO leads to a higher compressive strength due to the formation of calcium silicate hydrates (C–S–H) [29,34,35].

Blast-furnace slag (BFS) is, like fly ash, an industrial by-product, generated during iron and steel production in a blast furnace. According to ASTM C125-19 [36], blast-furnace slag can be classified as a nonmetallic product, consisting mainly of CaO, SiO_2_ and Al_2_O_3_. In comparison to fly ash, blast-furnace slag exhibits a higher amount of calcium. Therefore, C–S–H phases are one of the main products of alkali-activated blast-furnace slag [37,38,39].

Another common aluminosilicate source very low in calcium is metakaolin, a pozzolanic material obtained through the dehydroxylation of the clay mineral kaolinite at temperatures between 650 °C and 750 °C [37,40,41]. Novais et al. investigated the mineralogical and chemical composition of metakaolin and fly ash and determined the amorphous SiO_2_ and Al_2_O_3_ and the crystalline phases quartz, muscovite and anatase as the main components of metakaolin. In comparison to fly ash, the XRD pattern of metakaolin shows a higher amorphous content, indicated by the broad reflection signal between the 2θ angles 20° and 30° [42].

There are numerous types of alkaline activators available for the synthesis of geopolymer concrete. According to Chindaprasirt et al., these activators can be divided into three main groups [43]:

- caustic solutions: MOH;
- non-silicate salts of weakly acids: M_2_CO_3_, M_2_SO_3_, M_3_PO_4_, MF;
- silicates: M_2_O·nSiO_2;_
- aluminates: M_2_O·nAl_2_O_3;_
- aluminosilicates: M_2_O·Al_2_O_3·_(2-6)SiO_2;_
- salts of strong acids: M_2_SO_4._

The most commonly used activators for the synthesis of geopolymers are alkaline hydroxides, alkaline silicates or mixtures of both. In this context, the so-called water glasses (xSiO_3_·yM_2_O·nH_2_O; M = Na, K) are often chosen because they are believed to have a dual effect on the formation of geopolymers. They act as an alkaline activator and induce the formation of a high silica primary gel [44]. In general, the type of activator influences the properties of the synthesized geopolymer. For example Kubba et al. investigated the impact of curing temperatures and alkaline activators (sodium hydroxide, sodium silicate, mixture of both) on different properties of geopolymers based on granulated blast-furnace slag (GBFS), fly ash (FA) and palm oil fuel ash (POFA). The best results for a high compressive strength are achieved with a sodium silicate solution [45]. Baszczyski et al. investigated the different effects of sodium and potassium-based activators on the strength of geopolymers. They showed that, combined with siliceous fly ash, potassium-based activators provide better results than sodium-based activators [46].

## 2. Natural Fibers

**Figure 2:**
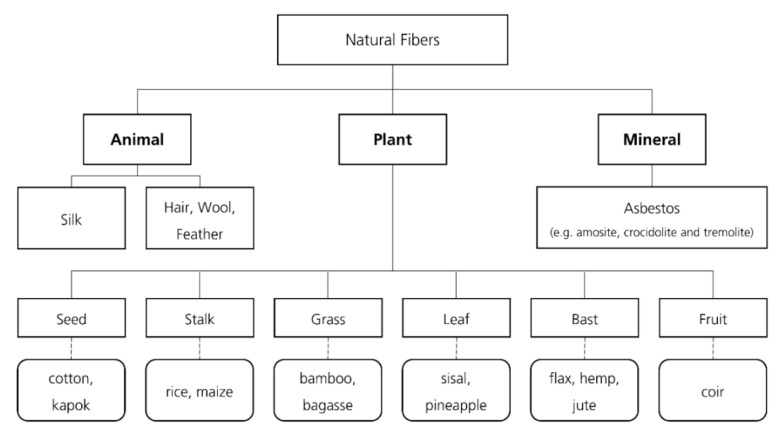
Classification of natural fibers according to Reference [47].

Natural fibers can, according to Bhattacharyya et al., subdivided into plant, animal and mineral fibers, as shown in Figure 2. Plant fibers are mainly composed of cellulose, hemicellulose, lignin and pectin [47]. In contrast, animal fibers consist of proteins, such as keratin or fibroin and, mineral fibers are mostly based on asbestos [47,48,49].

Primarily due to their beneficial properties, natural fibers have received much attention in the development of eco-friendly composite materials. The beneficial properties include low cost, low density, flexibility, renewability and recyclability [50]. Currently, several researchers are investigating bio-based building materials composed of geopolymer concrete and natural fibers, such as bamboo, cotton and hemp (Table 1) [47,48,49].

Tan et al. investigated the compatibility and the resulting flexural strength of metakaolin-based geopolymers with different types of wood (e.g., toona sinensis, fir, camphor) and non-wood fibers (e.g., bagasse, peanut shell, bamboo). The study showed, in most cases, slightly higher flexural strength values ranging from 5.3 to 9.1 MPa for the use of 5 wt. % wood fibers than for the use of 5 wt. % non-wood fibers ranging from 1.9 to 6.4 MPa [56]. Assaedi et al. manufactured fly ash-based geopolymer composites reinforced with different amounts of cotton (0, 1.4, 2.1, 2.8 and 4.1 wt. %) and flax fibers (0, 2.4, 3.0 and 4.1 wt. %). They reported an increase in flexural strength from 4.5 MPa to 23 MPa after the addition of 4.1 wt. % flax fibers. In the case of cotton fibers, the highest flexural strength is reached at a fiber content of 2.1 wt. %. Beyond this fiber content, the poor fiber–matrix interfacial bonding between the cotton fibers and the geopolymer matrix reduces the mechanical strength [50]. Sankar et al. studied geopolymer composites reinforced with 5 wt. % bamboo fibers. The average reported flexural strength was around 7.5 MPa. However, the pulled-out bamboo fibers on the scanning electron microscopy (SEM) images indicate a weak fiber matrix bonding [52]. Sá Ribeiro et al. also investigated geopolymer–bamboo composites. Their samples reach an average compressive strength of 23–30 MPa and an average flexural strength of 6–25 MPa after the addition of 5 wt. % bamboo fibers. In comparison with geopolymers without fibers, the addition of bamboo fibers leads to an increase in flexural strength and a decrease in compressive strength. The samples without fibers achieved a flexural strength of about 4.5 MPa and a compressive strength of 56 MPa [53]. Natali Murri et al. fabricated wool–geopolymer composites using metakaolin, potassium poly-silicate and potassium hydroxide. Their samples with 10 wt. % and 15 wt. % wool fibers achieved an average density of 1.0 g/cm^3^. For the wool–geopolymer composite with 10 wt. % fibers, the compressive strength and flexural strength were reported to be around 8.6 MPa and 5 MPa, respectively. The composites with 15 wt. % wool fibers reach a slightly lower compressive (8.3 MPa) and flexural (4 MPa) strength [55].

**Figure 3:**
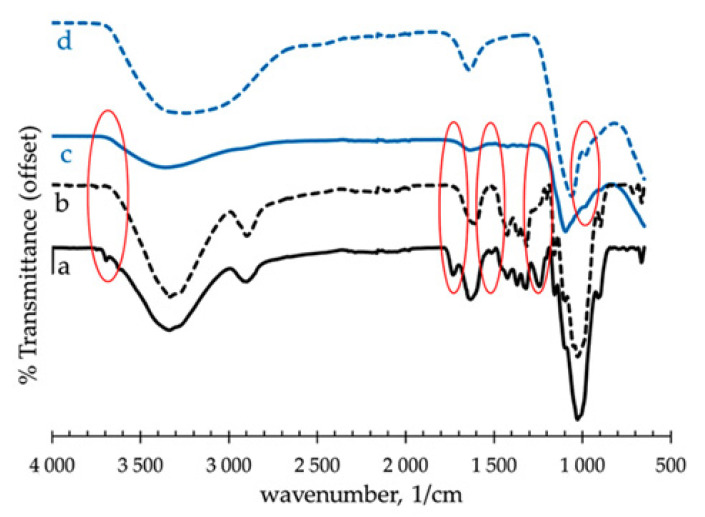
FTIR spectra of (**a**) untreated hemp; (**b**) NaOH-treated hemp; (**c**) NaOH + Al_2_(SO_4_)_3_ treated hemp; and (**d**) precipitate or residue from the spend solution of Al_2_(SO_4_)_3_ treatment. Reprinted from Reference [51] under open access license.

As the above-mentioned studies show, the strength of natural fiber-reinforced geopolymer composites is influenced by the type of the used plant fibers and strongly depends on the interfacial bonding between the binder and the fibers. This last aspect is one of the greatest challenges in the use of plant fibers in bio-based building materials. Therefore, Malenab et al. studied the influence of chemical treatment of abaca (hemp) fibers to improve the adhesion with the geopolymer matrix. In that study, different types of chemical treatments were used; (1) alkali treatment with NaOH, (2) aluminum sulfate (Al_2_(SO_4_)_3_) treatment and (3) alkali treatment with NaOH and afterward aluminum sulfate treatment. The FTIR results in Figure 3 indicate that an alkali treatment dissolves amorphous and hydrophobic components such as lignin, pectin and hemicellulose. Furthermore, the influence of chemical treatment on the mechanical properties and the interfacial bonding between the geopolymer matrix and fibers is investigated. The incorporation of hemp fibers primarily improves the flexural strength of geopolymers. Compared to geopolymers without fibers (2.8 MPa), the flexural strength was improved up to 5.5 MPa for geopolymers with untreated fibers and up to 7.3 MPa for geopolymers with treated fibers. The investigation of the microstructure by SEM indicates a better adhesion between the geopolymer matrix and treated fibers due to the formation of zeolite-like particles on the fiber surface. Nevertheless, the chemical treatment of natural fibers has to be improved, because some pull-out sites are suggesting an incomplete interfacial adhesion in some area [51]. These results also confirm the investigations of Zhou et al. on alkali-treated cotton fiber geopolymers. In comparison to untreated cotton fiber-based geopolymers, the compressive and flexural strengths of alkali-treated cotton fibers-based geopolymers can be improved by 4.8% and 11.5%, respectively [54].

### Effect of Nanoparticles on the Fiber–Matrix Adhesion

**Figure 4:**
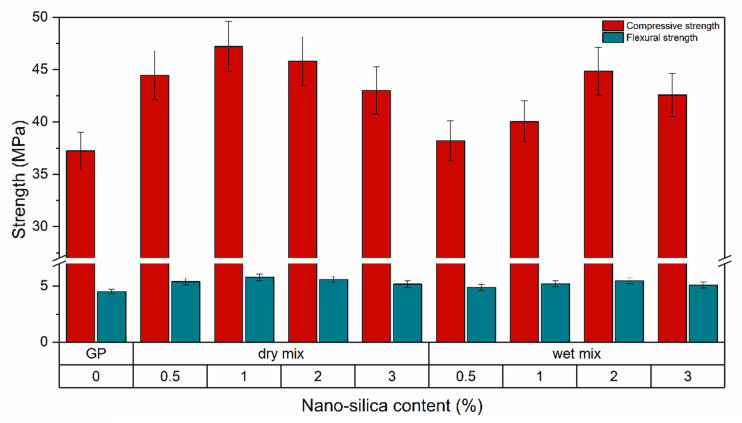
Influence of nano-sillica content on flexural and compressive strength of geopolymer samples according to Reference [14].

Recently, nanoparticles have received significant attention as an additive for geopolymer concrete. Nano-silica, nano-alumina and nano-clay are the most widely investigated nanomaterials to improve the mechanical properties of geopolymers [14,57,58,59,60,61]. Assaedi et al. investigated the influence of the mixing method (dry and wet mix) and the amount of nano-silica on the flexural and compressive strength. The reported compressive strength of their geopolymer samples increased under dry mixing conditions from 37.2 MPa without nanoparticles to 47.3 MPa after the addition of 1.0 wt. % nano-silica. Under wet mixing conditions, the highest compressive strength of 44.9 MPa was reached, with the addition of 2.0 wt. % nano-silica compared to 37.2 MPa for the control. The addition of 2.0 and 3.0 wt. % nano-silica under dry mixing conditions and 3.0 wt. % under wet mixing conditions leads again to a decrease in strength (Figure 4). The investigation of the flexural strength showed a similar development. Under dry mixing conditions, the flexural strength of the samples increased from 4.5 MPa without nanoparticles to 5.8 MPa after the addition of 1.0 wt. % of nano-silica. The highest flexural strength under wet mixing conditions had the value of 5.5 MPa and was reached with the addition of 2.0 wt. % nano-silica compared to 4.5 MPa for the control. The further addition of 2.0 and 3.0 wt. % nano-silica for dry mixing conditions and 3.0 wt. % for wet mixing conditions led again to a decrease in flexural strength [14]. However, the influence of nanoparticles on the mechanical properties of the final geopolymer depends on the particle size of the nanoparticles. Haruehansapong et al. reported the effect of the particle size of nano-silica on the compressive strength of cement mortar. The particle size directly affects the compressive strength; a smaller particle size leads to a lower compressive strength. According to the authors, the poor dispersion and the agglomeration of smaller particles is a possible reason for this behavior [62]. Therefore, the characterization of the particle size is one of the key challenges. Useful size information can be provided by different methods, such as small angle X-ray scattering (SAXS), SEM, single particle ICP-MS (sp–ICP–MS) and XRD [63].

**Figure 5:**
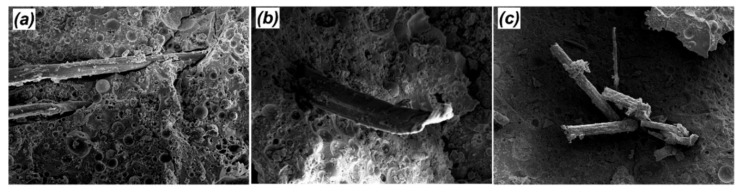
Scanning electron microscopy (SEM) images of flax fiber-reinforced geopolymer concrete; (**a**) geopolymer with flax fibers, (**b**) geopolymer with flax fibers and nano-silica (dry-mix) (**c**) geopolymer with flax fibers and nano-silica (wet-mix). Reprinted from Reference [57] under open access license.

The investigation of the effect of nanoparticles is not only crucial for the general improvement of geopolymer concrete but also has specific benefits for the development of fiber-reinforced geopolymer concrete. The combination of fibers and nanoparticles leads to reduced degradation of the fiber materials and results in better durability of the geopolymer composites [57,64,65,66,67]. Assaedi et al. investigated the influence of the nano-silica content on the mechanical properties and durability of a geopolymer concrete reinforced with flax fibers [57]. The average diameter of the silica nanoparticles is in the range of 18 to 25 nm. The flexural strength at four weeks increased from 23.0 to 30.5 MPa by the incorporation of 1 wt. % silica nanoparticles. Based on the SEM images (Figure 5), a positive effect on the durability of the flax fibers is also observed. In comparison to the flax fiber-reinforced geopolymer without nanoparticles (Figure 5a), the geopolymers with silica nanoparticles (Figure 5b,c) show a better adhesion between fiber and geopolymer matrix and the microstructure exhibits less unreacted fly ash particles. Consequently, the addition of nanoparticles leads to reduced degradation of the flax fibers [57]. Furthermore, Assaedi et al. studied the effect of nano-clay on the durability and mechanical properties of geopolymer concrete reinforced with flax fibers. They observed that the alkalinity of the geopolymer matrix is the main reason for the degradation of natural fibers and the addition of nanoparticles like nano-clay has a great potential to reduce this degradation [64]. This behavior is also confirmed by the research of Aly et al. Although they investigated flax fiber-reinforced waste-glass cement mortars, they reported that the addition of 2.5 wt. % nano-clay is an effective method to improve the durability of the flax fibers [66].

## 3. Geopolymer Foam Concrete

Foam or aerated concrete belongs to the class of lightweight concrete and can be defined according to ACI 523.2R-96 [68] as:

‘A lightweight product consisting of Portland cement and/or lime with siliceous fine material, such as sand, slag, or fly ash, mixed with water to form a paste that has a homogeneous void or cell structure. The cellular structure is attained essentially by the inclusion of macroscopic voids resulting from a gas-releasing chemical reaction or the mechanical incorporation of air or other gases (autoclave curing is usually employed).’ [68].

Accroding to EN 206 [69], foam or aerated concrete exhibits a density in the range of 800 to 2000 kg/m^3^, whereas the density of ordinary concrete ranges from 2000 to 2600 kg/m^3^. Due to the requirement of alternative, sustainable insulating materials with a low carbon footprint, the development of geopolymer foams has become more popular in the last few decades. Geopolymer foam concrete can be prepared by chemical or mechanical foaming or through the formation of syntactic foams. Table 2 summarizes examples of geopolymer foam concrete subdivided into chemical foaming, mechanical foaming and syntactic foams [69,70,71,72].

If geopolymer foam concrete should be considered as an alternative thermal insulation material, the properties of porosity, thermal conductivity and mechanical behavior must be investigated in order to improve the insulating properties. Thermal conductivity in particular is one of the essential properties of thermal insulating materials. It is usually investigated by using a heat-flow meter apparatus, which determines the thermal conductivity by measuring the heat-flow in a sample placed between two differently temperate plates [73,74]. Other authors use transient methods with needle probes [34] or hot discs [75]. The thermal conductivity of geopolymer foams strongly depends on the microstructure of the samples, because the microstructure of geopolymer foams has a significant influence on the properties of the material and is therefore of great interest in the characterization of samples. There are different methods in use to investigate the pore structure of geopolymer foams, which is usually of the closed type [50]. Therefore, a liquid penetration method such as the mercury intrusion porosimetry (MIP) method is, according to Ducman and Korat, limited to investigate the pore size distribution of geopolymer foams. At low pressure, it is unable to reach closed pores and at high pressure, it will break the cell walls between the pores [50]. Hence, MIP is often supplemented by other methods. Most authors use optical microscopy or scanning electron microscopy (SEM) of sample cross-sections and micro-computed tomography (μ-CT) as a non-destructive method to investigate the influence of different parameters such as the activator concentration or the amount and type of foaming agent on the porosity, pore size, pore size distribution and the morphology of pores incorporated in foamed geopolymers [10,32,76,77,78,79,80,81]. Furthermore, mechanical properties such as compressive and flexural strength are closely related to the porosity and microstructure of foamed geopolymers and much research has been focused on the effect of different parameters, such as the fly ash, metakaolin or foaming agent content, on these properties.

### 3.1. Chemical Foaming

For the chemical foaming method, a foaming agent is mixed with the other concrete ingredients to generate air voids which are due to a gas releasing reaction [34]. The most common foaming agents used for chemical foaming are hydrogen peroxide (H_2_O_2_) and aluminum powder (Al) [35]. In the case of aluminum powder, the voids are generated according to the following hydrogen releasing reaction [35,80,81].

2 Al + 2 OH^−^ + 6 H_2_O → 2 [Al (OH)_4_]^−^ + 3 H_2_ (1)

In contrast, hydrogen peroxide decomposes in basic media into water and oxygen; thus, the release of oxygen gas leads to the formation of air voids [82,84].

H_2_O_2_ → H_2_O + ½ O_2_ (2)

**Figure 6:**
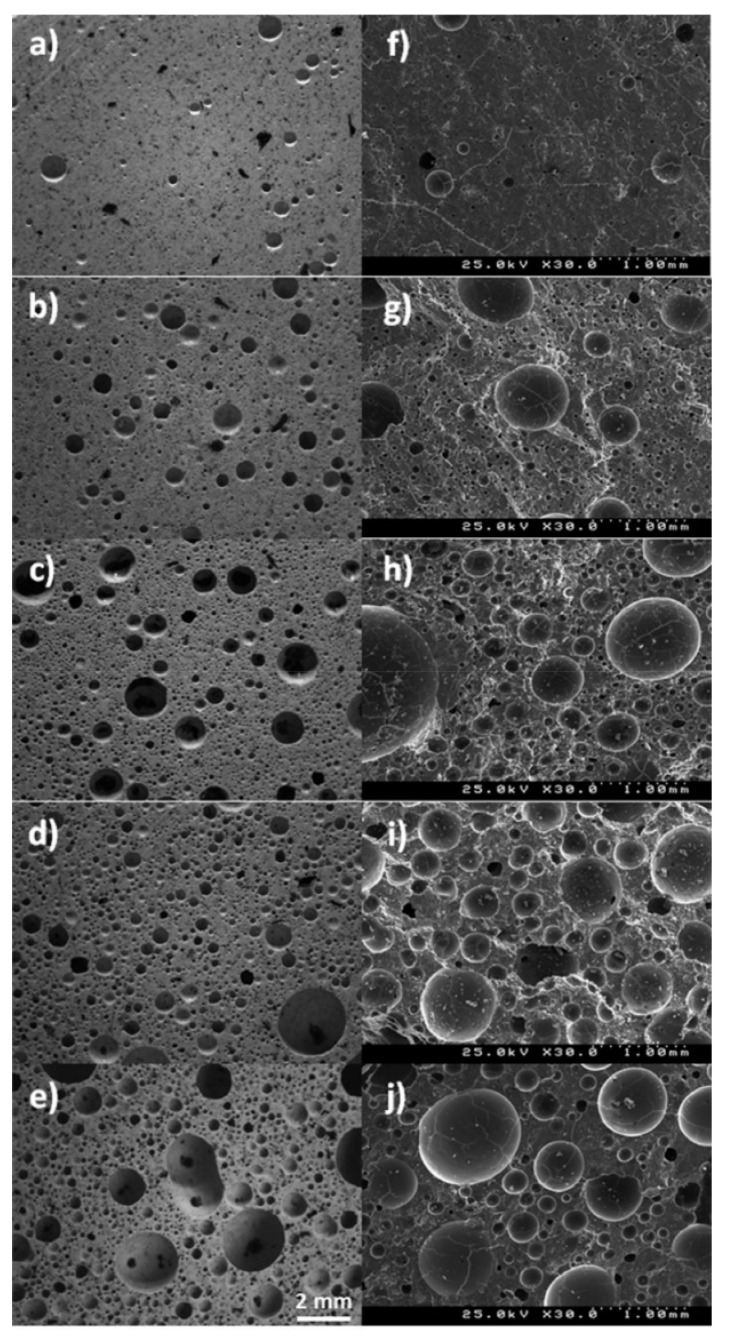
Optical microscopy and SEM characterization of fly ash geopolymers produced with different amounts of H_2_O_2_ as foaming agent ((**a**) and (**f**) 0.03 wt. %, (**b**) and (**g**) 0.15 wt. %, (**c**) and (**h**) 0.30 wt. %, (**d**) and (**i**) 0.90 wt. % and (**e**) and (**j**) 1.20 wt. %). Reprinted from Reference [42], © 2020, with permission from Elsevier.

Novais et al. synthesized geopolymer foams using fly ash as aluminosilicate sources and different amounts of H_2_O_2_ (0.03 to 1.2 wt. %) as a foaming agent. The analysis of the microstructure with optical and SEM microscopy (Figure 6) exhibits an increased porosity by increasing the H_2_O_2_ amount [82]. Consequently, the density, thermal conductivity and compressive strength decrease. [42]. Furthermore, Novais et al. have shown that the geopolymerization rate only depends on the concentration of alkali solution and the solid/liquid ratio. In contrast, the incorporation of the foaming agent does not influence the geopolymerization rate [82]. Petlitckaia et al. also investigated the influence of the hydrogen peroxide content on the microstructure and the mechanical properties of geopolymers. Besides the influence on the density, porosity, thermal conductivity and compressive strength, the H_2_O_2_ content also affects the pore size of the geopolymer foam. The pore size (500 to 3000 µm) increases by increasing the amount of H_2_O_2_ (0.25 to 2.5 wt. % *v*/*v*) [83].

A recent study by Hajimohammadi et al. investigated the regulation of chemical foaming to increase the porosity of fly ash-based geopolymer foams without increasing the amount of the foaming agent. The extent of the aluminum can be manipulated by changing the ratio of the alkali activators sodium hydroxide and sodium silicate. Therefore, a higher NaOH/Na_2_SiO_3_ ratio leads to a higher porosity [35].

**Figure 7:**
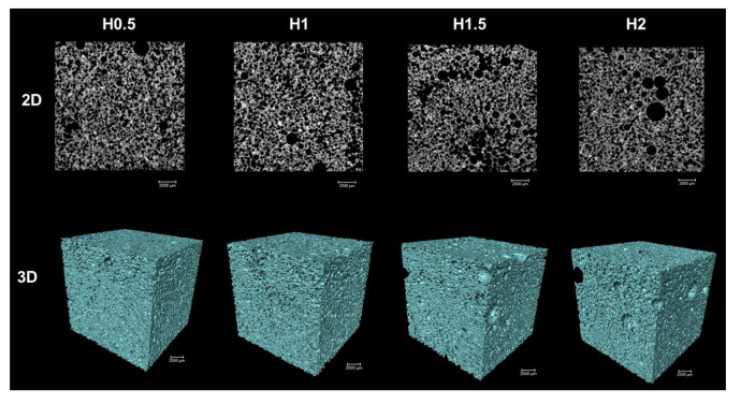
Micro-tomography images of geopolymer foams produced with H_2_O_2_ as a foaming agent. Reprinted from Reference [81], © 2020, with permission from Elsevier.

**Figure 8:**
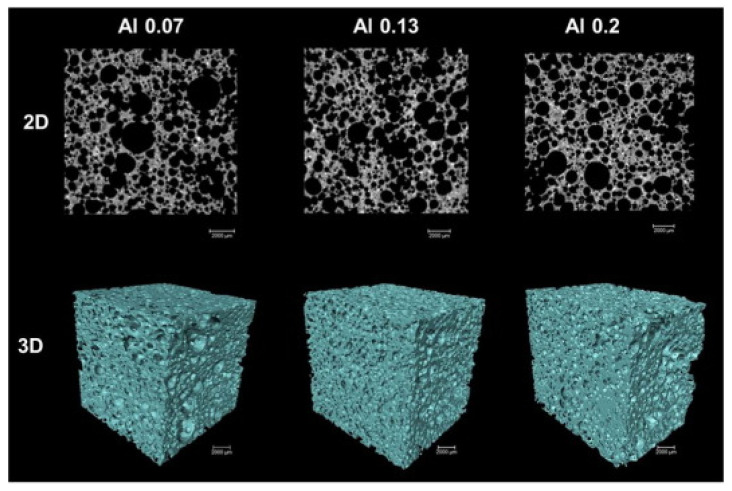
Micro-tomography images of geopolymer foams produced with Al powder as a foaming agent. Reprinted from Reference [81], © 2020, with permission from Elsevier.

Other authors like Ducman and Korat reported their research on fly ash-based geopolymer foam concrete, synthesized using hydrogen peroxide, as well as Al powder as a foaming agent. By comparing the results of the µ-CT measurements and the SEM analysis of both foaming agents, a difference in porosity and pore size is observed. They discovered that the use of H_2_O_2_ leads to smaller, more spherically shaped and more uniformly distributes pores than the use of Al-powder (Figure 7 and Figure 8). According to Ducman and Korat, the porosity of the geopolymer will increase up to a certain point with an increasing amount of foaming agent. At high amounts of foaming agent, the porosity of the material again decreases due to coalescence [81].

**Figure 9:**
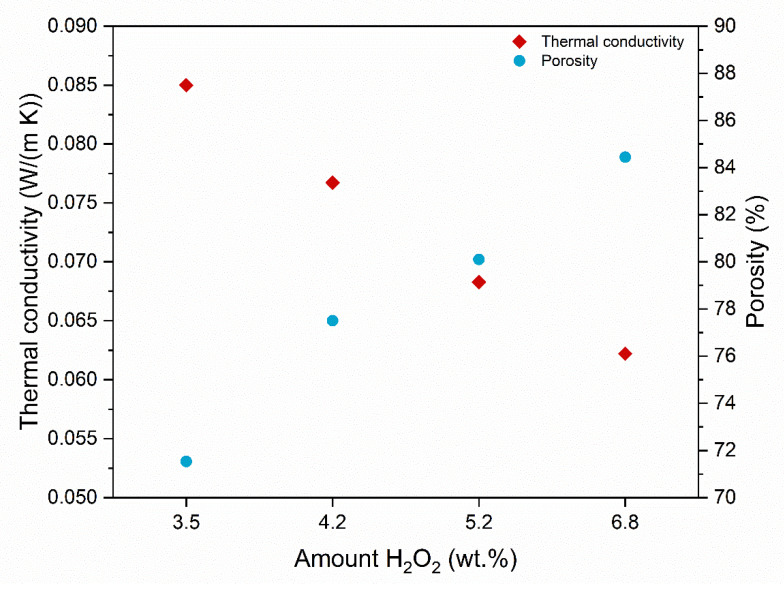
Porosity and thermal conductivity of geopolymers with different amounts of H_2_O_2_, according to Reference [73].

Wu et al. used a heat-flow meter apparatus to investigate the thermal conductivity of foamed geopolymers based on metakaolin, fly ash, and an alkaline activator containing sodium hydroxide, sodium water glass and distilled water. They used different amounts of H_2_O_2_ (3.5 wt. %, 4.2 wt. %, 5.2 wt. %, and 6.8 wt. %) as a foaming agent and stabilized the foam by adding calcium stearate. Wu et al. were able to lower the dry density of the material from 302 to 154 kg/m^3^ and increased the porosity from 71.8 to 84.5%, which resulted in a decreasing thermal conductivity from 0.0852 to 0.0622 W/(m K) (Figure 9) [73].

**Figure 10:**
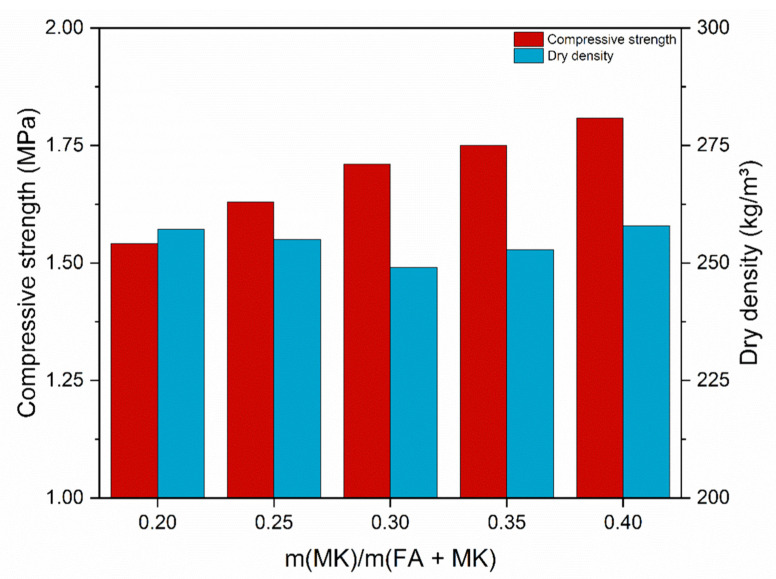
Influence of the metakaolin/fly ash ratio on dry density and compressive strength according to Reference [73].

**Figure 11:**
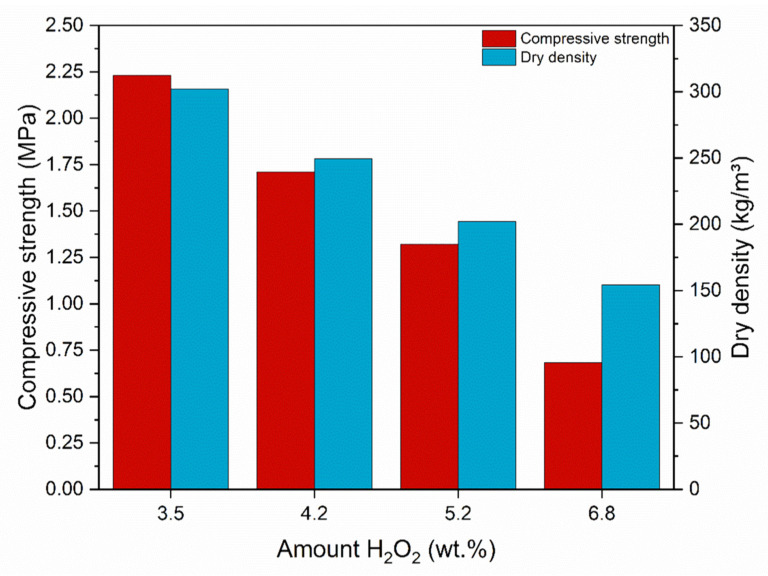
Influence of the amount of H_2_O_2_ on dry density and compressive strength according to Reference [73].

In this context, Wu et al. investigated the effects of the metakaolin/fly ash ratio on the compressive strength of geopolymers. They could demonstrate that an increase in the metakaolin/fly ash ratio also leads to an increase in the compressive strength of the samples without significantly effecting their dry density (Figure 10). The compressive strength increased from 1.54 MPa with a metakaolin/fly ash ratio of 0.2 to 1.84 MPa with a metakaolin/fly ash ratio of 0.4. Another parameter investigated which influences the compressive strength is the amount of foaming agent used. According to Wu et al., who used H_2_O_2_ as a foaming agent, the compressive strength of the geopolymers investigated decreases from 2.23 to 0.68 MPa with an increasing amount of H_2_O_2_ (3.5 to 6.8 wt. %), which consequently leads to a lower dry density of the samples (Figure 11) [73].

### 3.2. Mechanical Foaming

The mechanical foaming technique is subdivided into (1) mixed foaming and (2) pre-foaming. In the mixed-foaming method, foam is generated during the mixing process after adding a surfactant. In contrast, in the pre-foaming method, a pre-made foam is mixed with the concrete slurry [29,79].

Hajimohammadi et al. reported their research on geopolymer foam concrete, based on granulated blast-furnace slag (GBFS), synthesized by mixing a pre-made foam into the geopolymer mixture. Three different geopolymer mixtures with the same amount of pre-made foam were prepared to investigate the impact of the H_2_O/Na_2_O ratio and the Si/Al ratio on the properties of the final geopolymer foam. The XRD results of the different mixtures show that samples with a higher Si/Al ratio exhibit a lower amount of crystalline structures. However, an increasing crystallinity can be observed in samples with a higher H_2_O/Na_2_O ratio. The increased amount of water also leads to a poor homogeneity of pore size distribution, a lower thermal conductivity and reduces the early strength of the final geopolymer. In contrast, a lower Si/Al ratio leads to a higher strength [76].

**Figure 12:**
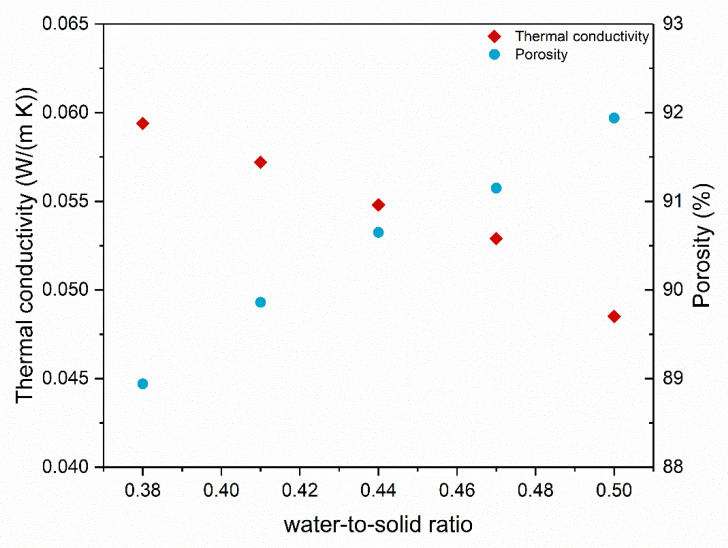
Porosity and thermal conductivity of geopolymers with different water-to-solid ratios according to Reference [86].

Another study of Hajimohammadi et al. investigates the effect of different amounts of xanthan gum (0.18 wt. %, 0.25 wt. %, and 0.45 wt. %) on the pore structure of fly ash-based geopolymers. The pre-made foam was prepared by mixing the foaming agent sodium dodecyl sulphate (SDS) and water. The increasing xanthan gum concentration reduces the collapse of the voids and leads to finer pores and, therefore, to a smaller pore size distribution compared to the control sample without xanthan gum. The compressive strength at 28 days is improved by up to 34% by a xanthan gum concentration of 0.45 wt. %. Furthermore, they reported a slight decrease in the thermal conductivity with an increasing amount of stabilizer. The samples with 0.45 wt. % xanthan gum showed a thermal conductivity of 0.21 W/(m K) compared to the control, with a thermal conductivity of 0.26 W/(m K) [34]. Cui and Wang studied the effect of the water-to-solid ratio on thermal conductivity. They could demonstrate that an increase in the water-to-solid ratio (Figure 12) leads to a reduction in the thermal conductivity from 0.06 W/(m K) to 0.048 W/(m K) [86].

### 3.3. Syntactic Foam

Besides the chemical and mechanical foaming technique, foam concrete can also be fabricated by embedding hollow spheres into a binding matrix. These so-called syntactic foams exhibit attractive mechanical and thermal properties. The most common hollow spheres, used for syntactic foams, are cenospheres, hollow glass micro-balls, hollow polymeric microspheres, and ceramic and metal hollow particles. Notably, the air- or inert gas-filled cenospheres are useful for the fabrication of syntactic foams due to their beneficial properties. The beneficial properties include lightweight, small size and spherical shape [77,78,84,85,87,88,89].

Hajimohammadi et al. manufactured geopolymer foam concrete using cenospheres, fly ash, ground granulated blast-furnace slag and sodium metasilicate. They reported achieving a density of 978 kg/m^3^, an average compressive strength of 17.5 MPa and thermal conductivity of 0.28 W/mK [77]. Wang et al. studied lightweight metakaolin-based geopolymeric composites using fly ash cenospheres. They achieved mixtures with 40 vol.% fly ash cenospheres, a density of 820 kg/m^3^, compressive strength of 36.5 MPa and thermal conductivity of 0.173 W/(m K) [84]. Shao et al. used hollow glass microspheres and fly ash to produce high-strength lightweight inorganic nonmetal materials (INM). They reported achieving 14.0 MPa in compressive strength for samples (580 kg/m^3^) with 50 wt. % hollow microspheres, 17.9 MPa in compressive strength for samples (641 kg/m^3^) with 40 wt. % hollow microspheres and 22.1 MPa in compressive strength for samples (782 kg/m^3^) with 30 wt. % hollow microspheres [85]. Zhang et al. made metakaolin-based geopolymers with two different types of hollow microspheres, namely, hollow phenolic microspheres and hollow glass microspheres. The density of the samples with hollow glass microspheres was about 1230 kg/m^3^, and the compressive strength was about 33.5 MPa. In contrast, the density of the samples with hollow phenolic microspheres was about 1280 kg/m^3^ and a reported compressive strength of about 22.5 MPa [78].

## 4. Development of Fiber-Reinforced Geopolymer Foam Concrete

Like aerated concrete, the properties of geopolymer foam concrete can also be improved using additives. Notably, fibers have shown a great impact on mechanical properties. [13,55,90]. However, nowadays, the most common fibers used in geopolymer foam concrete are syntactic fibers, such as polypropylene, polyvinyl alcohol or basalt fibers. Only a few researches focused on the development of natural fiber-reinforced geopolymer foam concrete, mainly based on abaca or hemp fibers. Table 3 summarizes examples of geopolymer foam concrete reinforced with fibers [13,88,91,92,93,94,95,96].

Rickard et al. manufactured fiber-reinforced metakaolin-based geopolymer foam concrete using 1 wt. % polypropylene fibers and different amounts of aluminum powder as a foaming agent (0.02, 0.04 and 0.06 wt. %). They reported that the addition of 0.06 wt. % aluminum powder leads to a density of 1.0 g/cm^3^ and a thermal conductivity of about 0.26 W/(m K). However, the high amount of foaming agent leads to an increased presence of large pores and therefore a compressive strength of 9.5 MPa. The addition of fibers to the un-foamed samples also leads to a reduction in compressive strength and density. The un-foamed geopolymer samples without fibers achieved a compressive strength of 54 MPa and a density of 1.61 g/cm^3^, whereas the samples with fibers achieved a compressive strength of 36 MPa and a density of 0.7 g/cm^3^ [91]. Masi et al. studied fly ash-based geopolymers that have been reinforced with 1 vol.-% polyvinyl alcohol (PVA) or basalt fibers and foamed using hydrogen peroxide and an unstated surfactant. They assume that the combination of hydrogen peroxide and the surfactant as foaming agents exhibited an easier control of density and porosity compared to foamed samples in which the foaming agents were used alone. The densities ranged from 1.2 to 1.7 g/cm^3^ for samples foamed with a surfactant, from 0.9 to 1.7 g/cm^3^ for samples foamed with hydrogen peroxide and from 0.7 to 1.7 g/cm^3^ for samples foamed with hydrogen peroxide and a surfactant. Furthermore, they reported the behavior under fire conditions of the fiber-reinforced and foamed geopolymer samples. The geopolymers reinforced with PVA fibers (thickness: 50 mm) show within 100 min a higher resistance under simulated fire conditions compared to the basalt fiber-reinforced geopolymers [92]. Lee et al. fabricated lightweight metakaolin-based geopolymers reinforced with carbon fibers (0.3, 0.5 and 0.7 wt. %) and foamed with hydrogen peroxide achieving densities of 415–431 kg/m^3^, thermal conductivities of 0.06–0.07 W/(m K) and reporting flexural strengths of 1.5–1.9 MPa [93]. Abdollahnejad et al. investigated the drying shrinkage and mechanical properties of fly ash-based geopolymer foams with different amounts of polypropylene (PP) fibers (0.2, 0.6, 1.0 and 1.4 vol. %) and foaming agent (0.2, 0.5 and 0.8 vol. %) as well as different sizes of PP fibers (6 mm and 20 mm). An increase in foaming agent up to 0.8 vol. % leads to an increased drying shrinkage up to 50% and degradation of the mechanical properties, whereas the addition of PP fibers with a length of 6 mm decreases the drying shrinkage by up to 45% and improves the mechanical properties. The maximum compressive and flexural (2.0 MPa) strength was registered for samples reinforced with 1.4 vol. % PP fibers [94].

Some researchers also use natural fibers such as hemp or abaca for the reinforcement of geopolymer foam concrete. Galzerano et al. investigated lightweight geopolymer concrete, reinforced with hemp fibers for use as insulating materials. The hemp-based geopolymer was synthesized by adding a hemp fiber grid to a slurry composed of metakaolin and sodium silicate. In order to obtain a geopolymer foam, a foaming agent composed of silicon metal powder and a mixture of vegetable surfactants was used. The SEM images show the fracture surface of the geopolymer foam reinforced with hemp fibers. The high alkaline pH of the geopolymer system leads to chemical treatment of the fibers and, therefore, strong adhesion between the geopolymer matrix and hemp fibers is observed. Higher magnifications in particular exhibit excellent interfacial bonding between the matrix and fiber due to an etching of the fiber surface [13]. Ngo et al. manufactured lightweight geopolymer concrete using hydrogen peroxide as foaming agent and abaca fibers for reinforcement. The compressive strength ranged from 19.56 to 36.85 MPa and the flexural strength values ranged from 2.145 to 6.254 MPa [95]. Triwulan et al. studied lightweight fly ash-based geopolymer concrete that had been reinforced with abaca fibers (0.4, 0.6 and 0.8 wt. % from the fly ash weight). They reported that the increase in abaca fibers up to 0.8 wt. % leads to an increase in compressive strength up to 3.05 MPa and a decrease in porosity. The total porosity value decreases from 61 to 57.5% with an increasing amount of fibers (0.4 to 0.8 wt. %) [96].

### Challenges in Developing Fiber-Reinforced Geopolymer Foam Concrete

Fiber-reinforced geopolymer foam concrete has the potential of being an alternative to conventional thermal insulation materials. Besides the research that has been conducted, the field of fiber-reinforced geopolymer foam concrete, especially with natural fibers, faces many challenges. Some of these challenges are listed below.

- The variable quality, composition and mechanical treatment of natural fibers make comparisons between research efforts difficult.
- The improvement in the interfacial bonding between the matrix and fiber to avoid pulled-out fiber.
- The improvement in the foam stability causes a large total area of foam, and the subsequent high surface energy leads to instability of the system.

**Figure 13:**
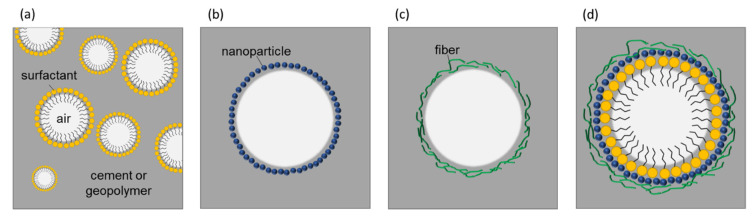
Foam stabilization via (**a**) a surfactant and Pickering emulsion formulated from (**b**) nanoparticles (**c**) fibers and (**d**) surfactant–fiber–nanoparticle (SFN) according to Reference [101].

Regarding the improvement in foam stability, one possible solution is presented by Krämer et al. They investigated a new approach for foam stabilization in concrete based on three phases. This three-phase-foam consists of a water/surfactant dispersion and is stabilized via nanoparticles by a Pickering emulsion [90,97]. In general, a Pickering emulsion (Figure 13), is a surfactant-based emulsion stabilized by solid particles such as nanoparticles or small fibers [98,99,100]. In the case of natural fiber-reinforced geopolymer foam concrete, a Pickering emulsion formulated using a surfactant, nanoparticles and fibers (Figure 13d) may be a useful foam stabilization method. 

## 5. Conclusions

The development of geopolymer foam concretes as sustainable thermal insulating materials is an important step toward more environmentally friendly buildings. Conventional thermal insulating materials, such as extruded or expanded polystyrene, mineral wool, foam or aerated concrete, have several disadvantages, including high-embodied energy, being based on non-renewable resources and high CO_2_ emissions. The present review describes the most important research findings related to fiber-reinforced geopolymer foam concrete and includes the topics natural fibers, foaming methods and recent efforts to reduce the fiber degradation in geopolymer concrete. The results show that the addition of natural fibers (e.g., abaca and hemp) improves the flexural and compressive strength as well as the thermal conductivity of foamed geopolymers. Furthermore, the combination of natural fibers and nanoparticles leads to higher durability of the fibers within the geopolymer matrix. However, these composites face many challenges, such as the improvement of the fiber–matrix adhesion or foam stability. Additionally, the variable quality, composition and mechanical treatment of natural fibers also make comparisons between research efforts difficult. Therefore, regarding the recent drive for sustainable alternatives, further studies will be required to replace the conventional thermal insulating materials with natural fiber-reinforced geopolymer foam concrete. Promising approaches are chemical treatment of fibers, combinations of natural fibers and nanoparticles and the use of adjusted foaming methods. Life cycle assessment and fire resistance of natural fiber-reinforced geopolymer foam concrete have not been reviewed in this paper, but they have been noted in some research studies and may represent challenges in the development of sustainable insulating materials based on natural fibers and geopolymer foam.

## Figures and Tables

**Table 1:** Overview of natural fibers used in geopolymer concrete.

Natural Fibers	Fiber Treatment	Aluminosilicate Source	Alkaline Activator	Curing Conditions		Results	Ref.
				T (°C)	t (h)		
Abaca (hemp) fibers	Alkali (NaOH) treatment and aluminum sulfate treatment	Fly ash	Sodium hydroxide and sodium silicate	75	24	Chemical treatment improves the interfacial bonding between the geopolymer and fiber.	[51]
Bamboo fibers	Water treatment	Metakaolin	Potassium hydroxide and potassium silicate	50	24	Bamboo fibers improve the flexural strength and strain.	[52]
Bamboo fibers	Alkali (NaOH) treatment and water treatment	Metakaolin	Sodium/potassium hydroxide and sodium silicate/potassium silicate	50	24	No difference in flexural strength between the fiber treatment methods.	[53]
Cotton fibers	Alkali (NaOH) treatment, PVA treatment and oil treatment	Fly ash	Sodium silicate	RT + 70	24 + 24	Alkali-treated cotton fibers improve the compressive and flexural strengths of the geopolymer.	[54]
Cotton and flax fibers		Fly ash	Sodium hydroxide and sodium silicate	Cured in the oven		Flax and cotton fibers improve the mechanical properties of geopolymers.	[50]
Hemp fiber grid		Metakaolin	Sodium silicate	40	24	Hemp fiber grids improve the mechanical properties.	[13]
Wool fibers		Metakaolin	Potassium hydroxide and potassium silicate	RT + 50	24 + 72	Wool fibers improve the flexural strength and fracture behavior.	[55]
Foona sinensis, Fir, Camphor, Black Locust, Eucalyptus, Korean pine, Poplar, Bagasse, Peanut shell, Wheat straw, Corn straw, Rice straw, Rice husk, Bamboo and Cassava straw		Metakaolin	Sodium hydroxide and sodium silicate	60	24	Wood fibers show a better compatibility with the geopolymer matrix than the non-wood fibers.	[56]

PVA = polyvinyl alcohol; RT = Room temperature.

**Table 2:** Overview of foaming methods for geopolymer concrete.

Foaming Method	Foaming Agent	Aluminosilicate Sources	Alkaline Activator	Curing Conditions			Results	Ref.
				T (°C)	T (h)	RH (%)		
Chemical foaming	Al powder	Fly ash	Sodium hydroxide and sodium silicate	60	24	n.r.	The ratio of alkaline activators impacts the extent of foaming.	[35]
Chemical foaming	Al powder	Fly ash	Sodium hydroxide	60	24	n.r.	Al powder delays the strength development and influences the gel formation.	[80]
Chemical foaming	Al powder	Metakaolin	Sodium hydroxide and sodium silicate				Increasing Al content leads to an increase in porosity and a decrease in thermal conductivity.	[74]
Chemical foaming	Al powder and H_2_O_2_	Fly ash	Sodium hydroxide and sodium silicate	70	24	n.r.	H_2_O_2_ as foaming agent leads to small pores and a compressive strength of 3.7 MPa (2.0 wt. % H_2_O_2_) Al powder as foaming agent leads to larger pores and a compressive strength of 3.3 MPa (0.2 wt. % Al powder).	[81]
Chemical foaming	H_2_O_2_	Metakaolin and fly ash	Sodium hydroxide and sodium silicate	40	24	65	NaOH concentration has an effect on compressive strength and thermal conductivity.	[82]
Chemical foaming	H_2_O_2_	Metakaolin and fly ash	Sodium hydroxide and sodium silicate	40	24	65	H_2_O_2_ content affects the physical properties (porosity, mechanical resistance and thermal conductivity).	[42]
Chemical foaming	H_2_O_2_	Metakaolin and fly ash	Sodium hydroxide and sodium silicate				Increasing H_2_O_2_-content leads to a decrease in compressive strength and thermal conductivity, and an increase in porosity.	[73]
Chemical foaming	H_2_O_2_ and different surfactants	Metakaolin	Sodium hydroxide and sodium silicate	RT + 60	10 + 24	n.r.	The surfactant influences the morphology and topology of the network and therefore the mechanical properties.	[83]
Chemical and mechanical foaming	H_2_O_2_ and SDS	Granulated blast-furnace slag and fly ash	Sodium hydroxide and sodium silicate	60	24	n.r.	Foam stabilizer enhances the pore size distribution and the pre-made foams are more stable.	[34]
Mechanical foaming	SDS	Granulated blast-furnace slag	Sodium hydroxide and sodium silicate	60	24	n.r.	A lower Si/Al ratio leads to higher strength, and a higher Si/Al ratio leads to a lower amount of crystalline structures.	[84]
Mechanical foaming	SDS	Granulated blast-furnace slag and fly ash	Sodium hydroxide and sodium silicate				Increasing XG concentration leads to an increase in compressive strength and a decrease in thermal conductivity	[34]
Mechanical foaming	Super-plasticizer	Fly ash	Sodium hydroxide and sodium silicate	RT + 60	24	n.r.	Higher compressive strength with heat curing (60 °C).	[29]
Syntactic foams	Cenospheres	Fly ash and ground granulated blast-furnace slag	Sodium metasilicate	RT	n.r.	n.r.	Strong bonding between cenospheres and the geopolymer matrix results in a compressive strength of 17.5 MPa at a density of 978 kg/m^3^ and a thermal conductivity of 0.28 W/(m K).	[77]
Syntactic foams	Fly ash cenospheres	Metakaolin	Potassium silicate	80	144	n.r.	Increasing the amount of cenospheres leads to a decrease in compressive strength, thermal conductivity and density.	[84]
Syntactic foams	Hollow glass microspheres	Fly ash	Sodium hydroxide and sodium silicate	60	24	n.r.	Increasing the amount of hollow glass microspheres leads to a decrease in density and compressive strength.	[85]
Syntactic foams	Hollow phenolic microspheres and hollow glass microspheres	Metakaolin	Sodium hydroxide and sodium silicate	40 + 60 + RT	2 + 24 + 144	n.r.	Increasing the amount of hollow microspheres leads to a decrease in compressive strength.	[78]

SDS = sodium dodecyl sulphate, n.r. = not reported, RT = room temperature, XG = Xanthan Gum.

**Table 3:** Overview of fiber-reinforced geopolymer foam concrete.

Fiber	Foaming Agent	Aluminosilicate Source	Alkaline Activator	Curing Conditions		Results	Ref.
				T (°C)	t (h)		
Carbon fibers	H_2_O_2_	Metakaolin	Potassium hydroxide and potassium silicate	90 + 40	24	Carbon fibers improve the flexural strength and thermal conductivity.	[93]
Polypropylene fibers	Mechanically prepared foam	Fly ash	Alkali Activator	28	24	The addition of PP fibers decreases the drying shrinkage and improves the mechanical properties.	[94]
Polypropylene fibers	Al powder	Metakaolin	Sodium hydroxide and sodium silicate	70	24	The addition of fibers decreases the density and compressive strength.	[91]
Polyvinyl alcohol and basalt fibers	H_2_O_2_ and surfactant	Fly ash	Sodium hydroxide and sodium aluminate	70	24	Geopolymers reinforced with PVA fibers show a higher resistance under simulated fire conditions compared to the basalt fiber-reinforced geopolymers.	[92]
Abaca fibers	H_2_O_2_	Fly ash	Sodium hydroxide and sodium silicate	RT	24	Abaca fibers improve the compressive strength.	[95]
Abaca fibers	Mecofix	Fly ash	Sodium hydroxide and Si_2_O_3_	60	6	An increasing amount of abaca fibers leads to an increase in compressive strength of the lightweight geopolymer concrete.	[96]
Hemp fibers	Si powder and mixture of vegetable surfactant	Metakaolin	Sodium silicate	40	24	Hemp fiber grids improve the mechanical properties.	[13]

PP = Polyproplyene, RT = room temperature.
